# Supplementary material for: Perceptions of burnout among public sector physicians in Sierra Leone: A qualitative study
Source: PLOS Glob Public Health. 2024 Sep 16;4(9):e0003739. doi: 10.1371/journal.pgph.0003739 (PMC11404797; doi:10.1371/journal.pgph.0003739)
Supplement: Attachment — Submitted filename: Response to Reviewers.docx [file pgph.0003739.s002.docx]

May 25, 2024

Dear Editors and Reviewers,

Thank you for taking the time to review our manuscript and provide valuable feedback. We appreciate your suggestions for improvement and have carefully considered each point raised.

Below, we provide a point-by-point response to address each comment from the reviewers and editorial office.

We hope you find our edits and clarifying statements in response to reviewer recommendations acceptable. We thank you for your important role in helping to improve the quality of our manuscript.

All my best,

Mohamed Jalloh, MBChB, MSc

Research Fellow

McMaster University

**Reviewers' comments:**

Reviewer's Responses to Questions

**Comments to the Author**

**Review Comments to the Author**

Please use the space provided to explain your answers to the questions above. You may also include additional comments for the author, including concerns about dual publication, research ethics, or publication ethics. (Please upload your review as an attachment if it exceeds 20,000 characters)

**Reviewer #1:**

I examined the article with great interest; it's intriguing. The authors have endeavoured to present a qualitative study of the problem of burnout that is of interest to the scientific community in the context of a country with limited resources.
There are aspects to which the authors can adhere in order to improve the quality of their article.
--------------------------------------------------------
1- In the ‘abstract’, it is preferable to underline these points:
+ add keywords, such as perception and contextualisation, to make it easier to characterise and index the study in bibliographic databases.

**Response:**

Thank you. We have added “**perception**” to the list of keywords. We have revised the manuscript to include this and have removed the word “**conceptualisation**” altogether.

---------------------
2- With regard to the ‘introduction’ section, a number of points could be highlighted:
+ improve the general data section on the subject with more references, which is limited. And in any case, more recent literature. Ideally, from 2 to 5 years ago.

**Response:**

Thank you. We have improved and expanded the ‘Introduction’ section with more recent references. (**See page 4 – 5**)

---------------------
3. In the ‘discussion’ section, the following points can be put forward to improve the quality of the article:
+ It is preferable to enrich the discussion with more references on the various themes raised by the study.

**Response:**

Thank you. We have improved and expanded the ‘discussion section with more recent references. (**See page 19-20**)

**Reviewer #2:** About the title.

The title of the article announces two components - objectives: conceptualization and doctors' perceptions, however, the article does not present conceptualization information in the results, in the discussion or in the conclusion.

In the introduction of the article the concept that served as the basis for this qualitative study is cited and in Study Design of methods it is said: “We adopted a pragmatic approach involving both deductive and inductive elements. However, we did not aim to produce a formal and generalizable theory. Then, in Data

Analysis: “Multiple strategies were used to enhance the validation of the data interpreted.(14) As we progressed with the coding, connections between categories emerged, facilitating a more theoretical coding approach. This method, paired with ongoing comparison, enabled a transition from descriptive to conceptual analysis. Consequently, we were able to construct a framework that guided further data sampling and coding, which enhanced our understanding of emerging themes and data saturation.” This description informs us how the researchers organized the sample, the interviews and the responses of the interviewees, for the analysis and presentation of the results, confirming that the qualitative study was focused on perceptions.

Due to the above, I suggest reviewing the title: “Conceptualization and perceptions of health workers on burnout in Sierra Leone: a qualitative analysis of public sector doctors”, I propose as an example: Qualitative study of Perceptions on burnout in the work of public sector doctors in Sierra Leone. 2022.

**Response:**

Thank you for this feedback. We have revised the title to accommodate this suggestion. (**See title page 1**)

**Revised Title:**

“Perceptions of Burnout Among Public Sector Physicians in Sierra Leone: A Qualitative Study”

**Reviewer's Comment:**
In the results I suggest checking:

**Physicians Recommendations for Change,** “Financial incentives and appropriate compensation for overtime work are essential for recognizing and motivating extra effort.” (...) It is crucial to implement strategies that guarantee the long-term viability of the recommendations outlined below.”

And in:

**Financial Incentives and Overtime Compensation:** which contains the researchers' analysis that appears in the article as: “Incentivizing overtime work can provide financial support and recognition for the additional efforts made by physicians, potentially enhancing morale and reducing burnout.”, because executing these statements would produce, that the monetary payment of overtime work the institutionalization of burnout, in contradiction of all the findings of the study.

In conclusion I propose to take into account in the writing:

The writing of the conclusion must compare the objectives and results based on the list of perceptions obtained from the interviewed doctors who describe explanations, demands and aspirations from their work experience.

**Response:**

The conclusion has been revised to compare the study's objectives and findings. It now clearly outlines how the identified themes—such as heavy workloads, resource constraints, and lack of institutional support—align with the study's aims and reflect the doctors' perceptions, demands, and aspirations. (**See pages 21 – 22**)

**Revised Conclusion:**

“In this study, we aimed to explore the experiences and perceptions of burnout among public-sector physicians in Sierra Leone. We found that organizational and relational factors, including heavy workloads, resource constraints, and lack of institutional support, significantly contribute to burnout. Detailed accounts from interviewed physicians highlighted the relentless nature of their work schedules, the emotional and professional toll of inadequate resources, and the urgent need for systemic changes to improve their work environment and overall well-being. To mitigate the effects of burnout, policymakers should adopt a multifaceted approach. This involves investing in the healthcare workforce to alleviate workload, providing adequate necessary resources to reduce stress and boost morale, and empowering workers through increased autonomy. Furthermore, promoting supportive and respectful work culture is of utmost importance. In resource-constrained settings, systemic reforms are necessary to improve working conditions, incentivize overtime, and develop strong support networks. These measures not only reduce the burden on healthcare providers, but also enhance their well-being and the quality of patient care they deliver.”

**Reviewer's Comment:**

The results of a qualitative study cannot be generalized as the perceptions of workers or doctors in the sector are restricted to the perceptions of the public sector doctors interviewed.

**Response:**

We acknowledge this important point. We have included a statement in the manuscript to clarify the limitations regarding the generalizability of our qualitative findings.

**Added Statement in Limitation Section:**

“It is important to note that the results of this qualitative study are based on the perceptions of the interviewed public sector physicians and may not be generalizable to all healthcare workers in Sierra Leone or other contexts. These findings provide valuable insights into the specific experiences of the participants, which can inform targeted interventions in similar settings.” (**See page 21)**

**Reviewer's Comment:**

Perceptions describe a situation resulting from a set of labor relations in a health system with limitations and scope. More than a risk, it is a result.

The risk is to maintain the situation until it is no longer temporary and becomes a characteristic of the institution or system that serves the population in conditions of poverty.

The social organization of medical work should not include monetary incentives to work overtime because it would negate the organizational and relational factors that produce job burnout.

**Response:**

Thank you for your insightful comment. We acknowledge that financial incentives and appropriate compensation for overtime work are important for recognizing and motivating physicians. We also agree that these measures alone may not guarantee the long-term viability of the recommendations. We also appreciate the reviewer’s concern regarding the potential institutionalization of burnout through the implementation of financial incentives for overtime work.

To address this, we have revised the manuscript to emphasize the importance of a comprehensive approach that includes both financial and non-financial strategies to support physicians and ensure sustainable improvements, and by highlighting the potential risks and emphasizing the need for a balanced approach that prevents the institutionalization of burnout. **(see page 20)**

This will be reflected in the revised text as follows:

**Revised Text:**

“Introducing monetary incentives for overtime within the social organization of medical work has broader implications. While financial incentives can provide immediate support and recognition,(42) it is crucial to implement these measures cautiously to avoid inadvertently contributing to the institutionalization of burnout.(43) A balanced approach that includes both financial and non-financial support, such as effective workload management, creation of supportive work environments, and provision of professional development opportunities. Addressing these areas comprehensively is crucial to ensure the long-term well-being of physicians.”
