# Supplementary material for: Perceptions of burnout among public sector physicians in Sierra Leone: A qualitative study
Source: PLOS Glob Public Health. 2024 Sep 16;4(9):e0003739. doi: 10.1371/journal.pgph.0003739 (PMC11404797; doi:10.1371/journal.pgph.0003739)
Supplement: Attachment — Submitted filename: Response to Reviewersv2.docx [file pgph.0003739.s003.docx]

August 18, 2024

Dear Editors and Reviewers,

Thank you for taking the time to review our manuscript and provide valuable feedback. We appreciate your suggestions for improvement and have carefully considered each point raised.

Below, we provide a point-by-point response to address comment from Reviewer #2 and editorial office.

We hope you find our edits and clarifying statements in response to reviewer recommendations acceptable. We thank you for your important role in helping to improve the quality of our manuscript.

All my best,

Mohamed Jalloh, MBChB, MSc

Research Fellow

McMaster University

**Reviewers' comments:**

Reviewer's Responses to Questions

**Comments to the Author**

1. If the authors have adequately addressed your comments raised in a previous round of review and you feel that this manuscript is now acceptable for publication, you may indicate that here to bypass the “Comments to the Author” section, enter your conflict of interest statement in the “Confidential to Editor” section, and submit your "Accept" recommendation.

Reviewer #1: All comments have been addressed

Reviewer #2: (No Response)

2. Does this manuscript meet PLOS Global Public Health’s [publication criteria](https://c05y1x9s.r.us-east-2.awstrack.me/L0/https:%2F%2Fjournals.plos.org%2Fglobalpublichealth%2Fs%2Fcriteria-for-publication/1/010f01914d8da464-3ca21a88-bb2c-418e-bef0-620a9fdda98d-000000/qesddxsNiGvwFcYS1p2rCdaVuvo=172)? Is the manuscript technically sound, and do the data support the conclusions? The manuscript must describe methodologically and ethically rigorous research with conclusions that are appropriately drawn based on the data presented.

Reviewer #1: Yes

Reviewer #2: Yes

3. Has the statistical analysis been performed appropriately and rigorously?

Reviewer #1: Yes

Reviewer #2: N/A

4. Have the authors made all data underlying the findings in their manuscript fully available (please refer to the Data Availability Statement at the start of the manuscript PDF file)?

The [PLOS Data policy](https://c05y1x9s.r.us-east-2.awstrack.me/L0/https:%2F%2Fjournals.plos.org%2Fglobalpublichealth%2Fs%2Fdata-availability/1/010f01914d8da464-3ca21a88-bb2c-418e-bef0-620a9fdda98d-000000/JOvLmS7ynxrMbaXn7kFovg6TW6o=172) requires authors to make all data underlying the findings described in their manuscript fully available without restriction, with rare exception. The data should be provided as part of the manuscript or its supporting information, or deposited to a public repository. For example, in addition to summary statistics, the data points behind means, medians and variance measures should be available. If there are restrictions on publicly sharing data—e.g. participant privacy or use of data from a third party—those must be specified.

Reviewer #1: Yes

Reviewer #2: Yes

5. Is the manuscript presented in an intelligible fashion and written in standard English?

PLOS Global Public Health does not copyedit accepted manuscripts, so the language in submitted articles must be clear, correct, and unambiguous. Any typographical or grammatical errors should be corrected at revision, so please note any specific errors here.

Reviewer #1: Yes

Reviewer #2: Yes

6. Review Comments to the Author

Please use the space provided to explain your answers to the questions above. You may also include additional comments for the author, including concerns about dual publication, research ethics, or publication ethics. (Please upload your review as an attachment if it exceeds 20,000 characters)

Reviewer #1: Perceptions of Burnout Among Public Sector Physicians in Sierra Leone: A Qualitative Study

PGPH-D-24-00567R1

Following improvements to the manuscript quality, we are confident that it is technically sound and should be accepted for publication. This is because it provides valuable information that can contribute to the understanding of the organisational structures and characteristics necessary for public sector doctors to flourish in a country with limited resources.

**Reviewer #2: I reviewed the abstract and the final version of the manuscript

Summary: Abstract
The title is changed correctly; however, the conclusions are recommendations of the researchers (of the authors of the article), it does not contrast the results of the research with the objectives of the research. It is striking to maintain in the conclusions “offer incentives for working overtime” when that is a minority expression among those interviewed.**

**Response:**

We thank the reviewer for their valuable feedback and the opportunity to improve our manuscript. In response, we have restructured the Results section in the Abstract to present our findings more logically - starting with workload issues and then progressing to emotional burden and coping mechanisms. The Conclusion now summarizes the key findings and provides specific recommendations for reforms, making it more action-oriented and aligned with the study’s objectives. Although "financial incentives and overtime compensation" emerged as a theme in our findings, we have de-emphasized it in the Conclusion, as it was a minority concern among the interviewees. (**See pages 2-3**)

**Revised Abstract Conclusion:**

“Within Sierra Leone's resource-constrained healthcare context, systemic reforms are necessary to address the root causes of physician burnout and improve patient care quality. Our findings suggest that implementing formal support structures, including counselling services and mentorship programs, is crucial. Improving working conditions through better resource allocation and infrastructure development is essential. Additionally, developing strategies to address the emotional burden of care, such as resilience training programs, could significantly impact physician well-being. By addressing these areas, reforms can enhance physician welfare, reduce burnout, and consequently improve the overall quality of patient care in Sierra Leone's public health sector.”

**Final manuscript

The title and text are maintained without the changes that are present in the summary.**

**Response:**

We are unsure which specific changes the reviewer is referring to, but we have revised the title to incorporate feedback from another reviewer. The new title aligns with the objectives and key aims of our study, as well as the revisions made based on all reviewers’ feedback. These changes are clearly reflected in the "Revised Manuscript with Tracked Changes" version of our manuscript.

7. PLOS authors have the option to publish the peer review history of their article ([what does this mean?](https://c05y1x9s.r.us-east-2.awstrack.me/L0/https:%2F%2Fjournals.plos.org%2Fglobalpublichealth%2Fs%2Feditorial-and-peer-review-process%23loc-peer-review-history/1/010f01914d8da464-3ca21a88-bb2c-418e-bef0-620a9fdda98d-000000/pzleIQu_yYYP5bGHgUwrl9dVZiE=172)). If published, this will include your full peer review and any attached files.

**Do you want your identity to be public for this peer review?** If you choose “no”, your identity will remain anonymous but your review may still be made public.

For information about this choice, including consent withdrawal, please see our [Privacy Policy](https://c05y1x9s.r.us-east-2.awstrack.me/L0/https:%2F%2Fwww.plos.org%2Fprivacy-policy/1/010f01914d8da464-3ca21a88-bb2c-418e-bef0-620a9fdda98d-000000/KfW5JYoEYShGj65iXTN_YpsQhBY=172).

Reviewer #1: **Yes:** Chadrack KABEYA DIYOKA

Reviewer #2: No

We have made our revisions directly on the attached revised version of our manuscript as requested.

We trust that our responses and subsequent revisions align with your expectations. We look forward to having our work published in PLOS Global Public Health and to disseminating the findings after publication.

Thank you for your time and consideration.

Sincerely

Mohamed Jalloh, MBChB, MSc

Research Fellow

McMaster University
